# Discovery of New Imidazole Derivatives Containing the 2,4-Dienone Motif with Broad-Spectrum Antifungal and Antibacterial Activity

**DOI:** 10.3390/molecules191015653

**Published:** 2014-09-29

**Authors:** Chunli Liu, Ce Shi, Fei Mao, Yong Xu, Jinyan Liu, Bing Wei, Jin Zhu, Mingjie Xiang, Jian Li

**Affiliations:** 1 Shanghai Key Laboratory of New Drug Design, School of Pharmacy, East China University of Science and Technology, 130 Mei Long Road, Shanghai 200237, China; 2 Radioimmunology and Clinical Laboratory, Luwan Branch, Ruijin Hospital, Shanghai Jiaotong University School of Medicine, Shanghai 200020, China; 3 Department of Laboratory Medicine, Ruijin Hospital, Shanghai Jiaotong University School of Medicine, Shanghai 200025, China; 4 Humanwell Healthcare (Group) Co, Ltd., 666 Gaoxin Road, East Lake High-Tech Development Zone, Wuhan 430075, China

**Keywords:** imidazole, 2,4-dienone, broad-spectrum, antifungal, antibacterial

## Abstract

A compound containing an imidazole moiety and a 2,4-dienone motif with significant activity toward several fungi was discovered in a screen for new antifungal compounds. Then, a total of 26 derivatives of this compound were designed, synthesized and evaluated through *in vitro* and *in vivo* antifungal activity assays. Several compounds exhibited improved antifungal activities compared to the lead compound. Of the derivatives, compounds **31** and **42** exhibited strong, broad-spectrum inhibitory effects toward *Candida* spp. In particular, the two derivatives exhibited potent antifungal activities toward the fluconazole-resistant isolate *C. albicans* 64110, with both having MIC values of 8 µg/mL. In addition, they had significant inhibitory effects toward two Gram-positive bacteria, *Staphylococcus aureus* UA1758 (compound **31**: MIC = 8 µg/mL; compound **42**: MIC = 4 µg/mL) and *Staphylococcus epidermidis* UF843 (compound **31**: MIC = 8 µg/mL; compound **42**: MIC = 8 µg/mL). The results of an animal experiment indicated that both compounds could improve the survival rate of model mice infected with ATCC 90028 (fluconazole-susceptible isolate). More importantly, the two compounds exhibited notable *in vivo* effects toward the fluconazole-resistant *C. albicans* isolate*,* which is promising with regard to the clinical problem posed by fluconazole-resistant *Candida* species.

## 1. Introduction

Infections caused by bacteria and fungi lead to diseases and an enormous social burden as millions of people are infected by bacteria and fungi every year worldwide. Therefore, a large number of antimicrobial drugs have been listed, which play an important role in treating infections [1]. As the need for antifungal intervention has increased, so too has the prevalence of resistance [2]. With the irrational use of antibiotics, the resistance of microorganisms has become a very serious clinical problem. Growing antifungal resistance poses the threat that there will be no available drugs for the treatment of common infections in the future [3], so there is an urgent need for the discovery of new compounds with antibacterial and antifungal activities [4], especially those with mechanisms of action that are distinct from the well-known classes of antifungal agents [5,6,7].

The development of derivatives based on heterocyclic scaffolds is a fast emerging subject in medicinal chemistry. Azole compounds in particular play a remarkably important role in the field of medicinal chemistry. A great deal of azole-based antibacterial and antifungal agents have been extensively studied as drug candidates, and some of them have been used in the clinic, for instance itraconazole, fluconazole, posaconazole and voriconazole, which suggests the great development value of azole compounds [8]. However, the reduced susceptibility of *Cryptococcus neoformans* to fluconazole has been reported in a number of sub-Saharan countries including Kenya, Uganda, Rwanda and South Africa [9]. As a result, there is an unmet need for the development of new azole antifungals.

**Figure 1:**
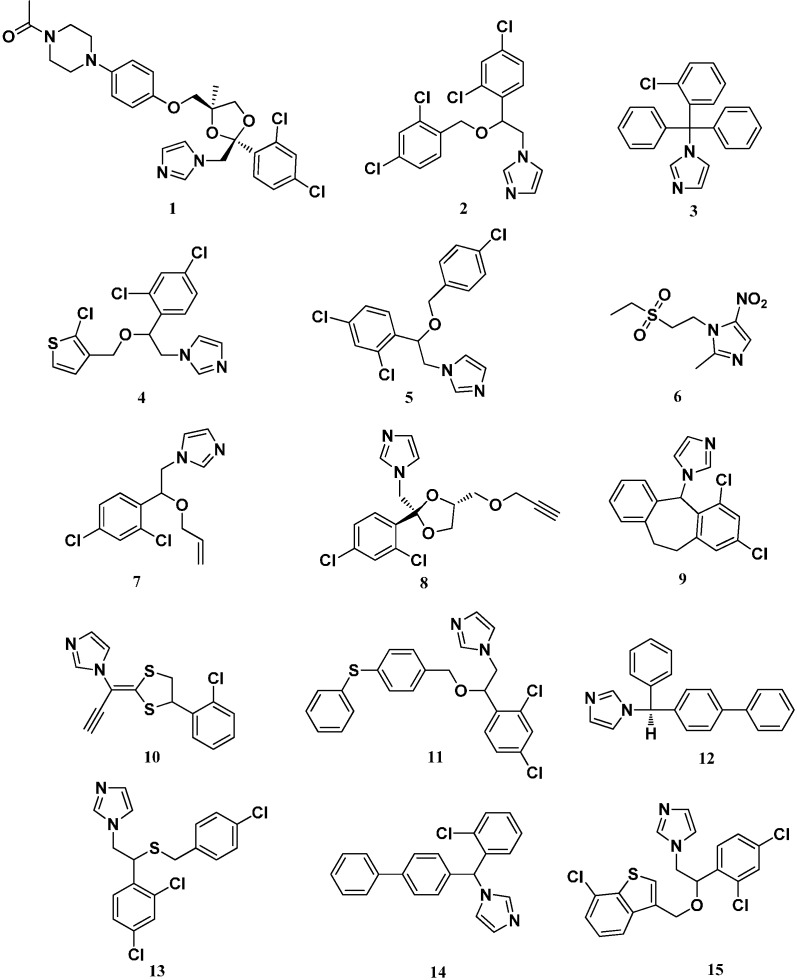
Marketed imidazole drugs.

Imidazole compounds containing two nitrogen atoms in a five-membered aromatic azole ring have received special attention in recent years. As reported, imidazole rings are widely employed as spin-trapping species in the interesting application of designing drugs with neuroprotective activity [10,11]. These imidazole-based derivatives also have several favorable properties such as excellent bioavailability, good tissue penetrability and permeability and a relatively low incidence of adverse and toxic effects, which suggests that they have considerable development potential in chemistry, materials science and medicinal chemistry [12]. Their extensive applications, especially as antifungal agents, are frequently investigated, and this has become one of the most active areas in antifungal drug development. Many imidazole-based derivatives have been marketed as antifungal drugs such as ketoconazole (**1**), miconazole (**2**), clotrimazole (**3**), tioconazole (**4**), econazole (**5**), tinidazole (**6**), enilconazole/imazalil (**7**), parconazole (**8**), eberconazole (**9**), lanoconazole (**10**), fenticonazole (**11**), bifonazole (**12**), sulconazole (**13**), lombazole (**14**), and sertaconazole (**15**) (Figure 1) [13,14,15,16,17,18,19], which indicates their large development value and broad potential as antifungal agents.

**Figure 2:**
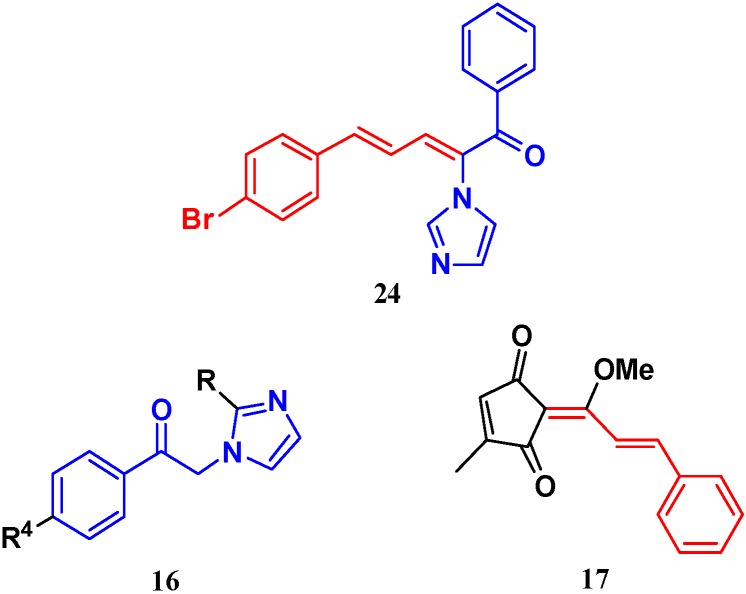
The structural scaffold of the lead compound synthesized in this study **24** and structurally similar compounds **16**–**17** reported in the literature.

The imidazole ring has been demonstrated to be a versatile core of many biologically active molecules, especially those with antifungal properties. Thus, we screened the imidazole-based compounds in our inventory. As a result, compound **24** in Figure 2 was discovered in our screening to exhibit antifungal activity.

The MIC values of this compound toward *Candida albicans* ATCC 90028, *Candida glabrata* 923, *Candida glabrata* 168 and *Candida parapsilosis* 27 were 2, 4, 4 and 16 µg/mL, respectively, but this compound was found to be inactive against *Candida albicans* 205, *Candida albicans* 64110, *Candida krusei* ATCC 6528, and *Candida tropicalis* 657. Considering the biological potency and synthetic accessibility of compound **24**, it has the potential to serve as a reasonable lead compound for further development.

From the viewpoint of a medicinal chemist, the chemical structure of this compound contains two distinctive moieties: a 2-(1*H*-imidazol-1-yl)-1-phenylethanone moiety and an (*E*)-buta-1,3-dien-1-ylbenzene moiety (**24** in Figure 2). A literature survey indicates that compounds containing such moieties exhibit antifungal activities. For example, a number of 2-(1*H*-imidazol-1-yl)-1-phenylethanone derivatives (for example **16** in Figure 2) exhibit powerful activities against *C. albicans* and *P. chrysogenum*, but moderate activity against *A. niger* at a concentration of 10 µg/mL [20]. In addition, (*E*)-2-((*E*)-1-methoxy-3-phenylallylidene)-4-methylcyclopent-4-ene-1,3-dione (**17** in Figure 2) exhibited varying degrees of antifungal activity against fungi *C. albicans* ATCC 90028 (2.08 µg/mL), *C. neoformans* ATCC 90113 (8.33 µg/mL) and *A. fumigatus* ATCC 90906 (>20 µg/mL) [21]. Given these facts, it is reasonable to expect that a compound combining these two moieties may have antifungal activities. Therefore, the active compound identified in our screening, compound **24**, is worthy of further development. Based on this information, in this study, we designed, synthesized and evaluated a series of derivatives **21**–**46** of compound **24** and evaluated their activities as antifungal and antibacterial agents.

## 2. Results and Discussion

### 2.1. Chemistry

The synthetic methods used to prepare compound.

**Scheme 1:**
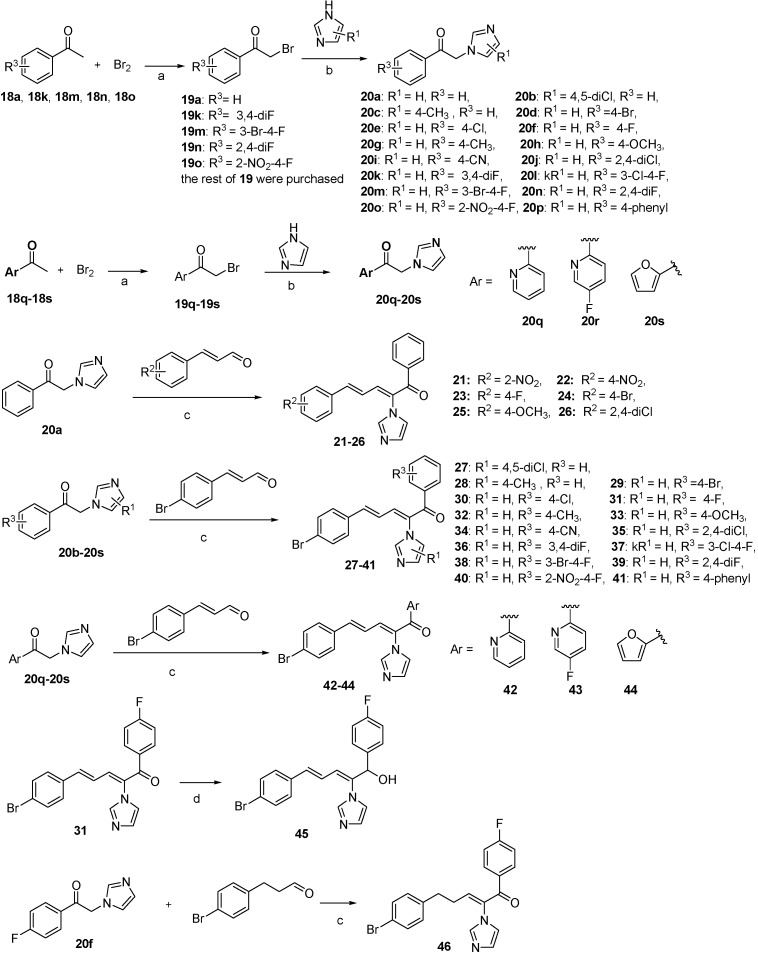
The synthetic routes used to prepare compounds **21**–**46**.

**21**–**46** are illustrated in Scheme 1. The non-commercial intermediates (compounds **19a**, **19k**, **19m**, **19n**, **19o**, **19q**, **19r**, **19s**) were synthesized by irradiating the appropriate acetophenone in the presence of Br_2_ at a power of 250 W for 5 h (Scheme 1). Then, compound **20**, the key intermediate, was obtained through the reaction of intermediate **19** with imidazole. Finally, various aldehydes were reacted with the corresponding intermediate **20** to produce the target compounds **21**–**44** and **46** via a Knoevenagel reaction. There was an interconversion between the *E* and *Z* isomers in the reaction solvent, which complicated the purification of the products. The ultimate solution was to purify the compounds by recrystallization from a mixed solvent of ethyl acetate and petroleum ether (v/v, 1/70) after silica gel column chromatography. The two-dimensional hydrogen spectrum suggested that the obtained compounds had only the *Z-*configuration.

To investigate the influence of the carbonyl moiety, compound **45** was also synthesized by reducing compound **31** with sodium borohydride, as shown in Scheme 1. All compounds were fully analyzed and characterized by ^1^H nuclear magnetic resonance (NMR) and high-resolution mass spectrometry (HRMS) before biological evaluation.

*Reagents and conditions*: (a) Br_2_, glacial acetic acid, 48% hydrogen bromide, 250 W, 5 h; (b) triethylamine, tetrahydrofuran, r.t., overnight; (c) piperidine, glacial acetic acid, toluene, 75 °C, 4 h; (d) NaBH_4_, anhydrous methanol, r.t.

### 2.2. Antifungal Susceptibility Assay

All of the synthesized compounds were evaluated using *in vitro* antifungal activity assays against *Candida albicans* ATCC 90028, *Candida albicans* 205, *Candida albicans* 64110, *Candida krusei* ATCC 6528, *Candida tropicalis* 657, *Candida glabrata* 923, *Candida glabrata* 168 and *Candida parapsilosis* 27 (Table 1). The minimum inhibitory concentrations (MICs) of 26 compounds and fluconazole (Pfizer Pharmaceuticals, Shanghai, China) for the tested isolates were determined based on the standard guidelines described in the Clinical and Laboratory Standards Institute document M27-A3 using the microdilution reference method.

The MICs of all antifungal agents against the tested strains of *Candida* spp. determined by broth microdilution are listed in Table 1. The MICs of most compounds were higher than those of fluconazole, while compounds **31** and **42** exhibited more potent inhibitory effects on the tested *Candida* spp. In particular, the two derivatives showed potent antifungal activity toward the fluconazole-resistant isolate *C. albicans* 64110, with a MIC value of 8 µg/mL, which was a relatively excellent result.

### 2.3. Conclusion of the Structure-Activity Relationship

SAR of this class of compounds has been well studied through variation of the styryl, imidazole and benzoyl moieties, as shown in Table 1. First, the substituents on the A-ring were varied to improve the antifungal activity of the compounds. Thus, the first batch of compounds (**21**–**23**, **25**, **26**) was synthesized by replacing the 4-Br (**24** in Figure 2) with 4-NO_2_, 2-NO_2_, 4-F, 4-OCH_3_, and 2,4-diCl. Unfortunately, all of the resulting derivatives exhibited weaker antifungal activities than the lead compound **24**. Both 2-substitution (**22**, **26**) and electron-donating groups (**25**) decreased the antifungal activity.

With the second batch of compounds (**27** and **28**), the effect of the imidazole moiety was investigated. The antifungal activities were largely abolished when the imidazole was replaced with 4-methyl-1*H*-imidazole or 4,5-dichloro-1*H*-imidazole.

For the third batch of compounds, sixteen compounds (**29**–**44**) were obtained and eight (**30**, **31**, **36**, **39**, **42**, **43**, **45**, **46**) of them exhibited noticeable antifungal activities. Among them, compounds **31** and **42** exhibited broad-spectrum antifungal activities with MIC values of 0.5–8 µg/mL and 2–32 µg/mL, respectively. Replacement of benzoyl with *p-*fluorobenzoyl or pyridin-2-yl-formyl was apparently beneficial with respect to their antifungal activities. In particular, 4-fluoro derivative **31** exhibited potent antifungal activity toward the fluconazole-resistant isolate *C. albicans* 64110, with a MIC value of 8 µg/mL. Increasing the electronegativity of the substituent (**34**) or the volume occupied by it (**29**, **30**, **41**) and introducing electron-donating groups (**32**, **33**) or second substituents (**35**–**40**) resulted in relatively poor inhibitory activities. The results above indicated that substitution of fluoro in the *para* position of the phenyl group was essential for maintaining the antifungal activity.

Furthermore, the antifungal activities of compounds **45** and **46** were also explored. Both of them exhibited a significant decrease in activity, which suggested that the conjugated double bonds and carbonyl were important for the activity.

### 2.4. Antibacterial Susceptibility Assay

The antibacterial activities of the two compounds (compounds **31** and **42)** with excellent antifungal activities were also evaluated by antibacterial activity assays. The minimum inhibitory concentrations (MICs) of the two compounds were determined based on standard guidelines described in the Clinical and Laboratory Standards Institute document M27-A3 using the broth microdilution reference method. The results are shown in Table 2. Among the tested strains, the two compounds were effective toward two Gram-positive bacteria, *Staphylococcus aureus* UA1758 (compound **31**: MIC = 8 µg/mL; compound **42**: MIC = 4 µg/mL) and *Staphylococcus epidermidis* UF843 (compound **31**: MIC = 8 µg/mL; compound **42**: MIC = 8 µg/mL).

**Table 1:** Chemical structures of compounds **21**–**46** and their antifungal activities. 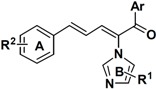

Compd.	R^1^	R^2^	Ar	Antifungal Activity MIC (μg/mL)							
				*C. albicans* ATCC 90028	*C. albicans* 205	*C. albicans* 64110	*C. krusei* ATCC 6528	*C. tropicalis* 657	*C. Glabrata* 923	*C. Glabrata* 186	*C. parapsilosis* 27
**21**	H	4-NO_2_	phenyl	>128	>128	>128	>128	>128	8	16	>128
**22**	H	2-NO_2_	phenyl	>128	>128	>128	>128	>128	>128	>128	>128
**23**	H	4-F	phenyl	32	>128	>128	>128	>128	>128	>128	>128
**24**	H	4-Br	phenyl	2	>128	>128	>128	>128	4	4	16
**25**	H	4-MeO	phenyl	>128	>128	>128	>128	>128	>128	>128	>128
**26**	H	2,4-diCl	phenyl	>128	>128	>128	>128	>128	>128	>128	>128
**27**	4,5-diCl	4-Br	phenyl	>128	>128	>128	>128	>128	>128	>128	>128
**28**	4-CH_3_	4-Br	phenyl	>128	>128	>128	>128	>128	>128	>128	>128
**29**	H	4-Br	4-Br-phenyl	8	>128	>128	>128	2	4	4	2
**30**	H	4-Br	4-Cl-phenyl	2	>128	>128	32	4	8	8	>128
**31**	H	4-Br	4-F-phenyl	0.5	4	8	8	2	2	2	4
**32**	H	4-Br	4-Me-phenyl	2	>128	>128	>128	>128	>128	>128	>128
**33**	H	4-Br	4-MeO-phenyl	>128	>128	>128	>128	>128	>128	>128	>128
**34**	H	4-Br	4-CN-phenyl	2	>128	>128	>128	4	1	1	4
**35**	H	4-Br	2,4-diCl-phenyl	2	>128	>128	>128	2	>128	>128	4
**36**	H	4-Br	3,4-diF-phenyl	2	>128	>128	32	16	8	8	16
**37**	H	4-Br	3-Cl-4-F-phenyl	>128	>128	>128	>128	>128	32	32	>128
**38**	H	4-Br	3-Br-4-F-phenyl	>128	>128	>128	>128	>128	>128	>128	>128
**39**	H	4-Br	2,4-diF-phenyl	0.5	>128	64	64	16	32	32	8
**40**	H	4-Br	2-NO_2_-4-F-phenyl	8	>128	>128	>128	>128	>128	>128	>128
**41**	H	4-Br	1,1'-biphenyl	>128	>128	>128	>128	>128	>128	>128	>128
**42**	H	4-Br	pyridin-2-yl	4	8	8	32	2	4	8	8
**43**	H	4-Br	5-F-pyridin-2-yl	4	32	64	8	2	64	64	1
**44**	H	4-Br	furan-2-yl	>128	>128	>128	>128	>128	>128	>128	>128
**45**	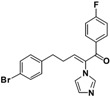			8	128	128	128	128	128	128	32
**46**	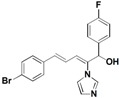			0.25	64	128	16	8	32	32	8
Fluconazole				0.5	8	>128	32	16	32	32	1

**Table 2:** The antibacterial activity of **31** and **42**.

Compd.	Antibacterial Activity MIC (μg/mL)							
	*Enterococcus faecalis* UA257	*Staphylococcus aureus* UA1758	*Staphylococcus epidermidis* UF843	*Klebsiella pneumonia* UF222	*Escherichia coli* UA45	ESBL-Producing *Escherichia coli*	*Pseudomonas aeruginosa* UA1024	*Acinetobacter baumannii* UA1037
**31**	>128	8	8	>128	>128	>128	>128	>128
**42**	>128	4	8	>128	>128	>128	>128	>128
Amikacin	n.t.^a^	n.t.^a^	n.t.^a^	2	8	3	3	>256
Cefoperazone	n.t.^a^	n.t.^a^	n.t.^a^	8	12	8	6	32
Vancomycin	1.5	0.75	2	n.t. ^a^	n.t. ^a^	n.t. ^a^	n.t. ^a^	n.t. ^a^
Erythromycin	2	>256	24	n.t. ^a^	n.t. ^a^	n.t. ^a^	n.t. ^a^	n.t. ^a^

^a^ n.t. = Not tested.

### 2.5. Antifungal Activity in Vivo

**Figure 3:**
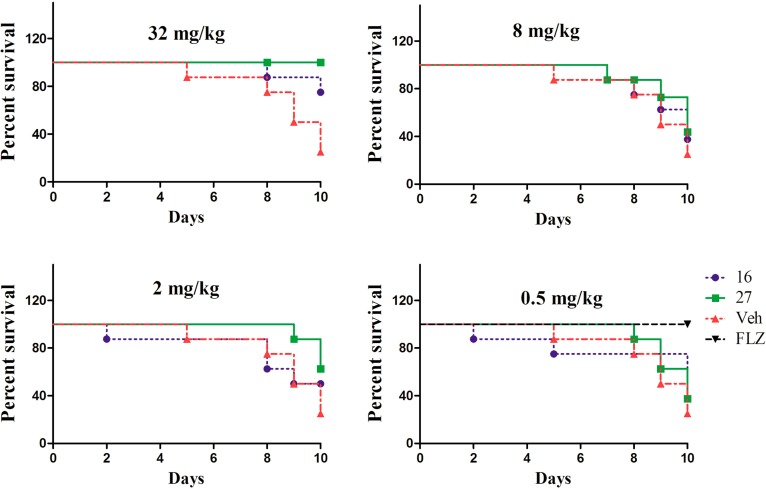
Survival rate of mice after injection with *C. albicans* ATCC 90028. FLZ stands for fluconazole and indicates a statistically significant difference compared to the control group. The four tested dosages of **31** and **42** were 32, 8, 2 and 0.5 mg/kg. The dosage of fluconazole was 0.5 mg/kg.

As compounds **31** and **42** demonstrated excellent antifungal activities *in vitro* and were more potent than fluconazole, their *in vivo* activities were investigated in the further studies. In these studies, a mouse model of systemic candidiasis was used. First, the toxicities of these compounds in the mice were explored. As the structures of the two compounds were similar, we only assessed the toxicity of compound **31**. Each group of mice was given compound **31** by intragastric administration consecutively with dosages of 32, 16 or 8 mg/kg once a day. After five continuous days of drug administration, nothing abnormal was detected. Therefore, the further studies could be continued under these dosages. As shown in Figure 3, after a prolonged time of injection of ATCC 90028 (fluconazole-susceptible isolate), the mice in the control group died gradually, while the survival rates of mice treated with compound **31** or **42** increased.

Compound **42** exhibited particularly excellent antifungal activity *in vivo*; all mice treated with 32 mg/kg of **42** survived to day 10. However, compared with the reference drug fluconazole, the effects of the two compounds were weak, which was not consistent with the *in vitro* assay results. This discrepancy may be due to the low hydrophilicity of compounds **31** and **42**. In future studies, we will focus on improving the hydrophilicity of these compounds to enhance their *in vivo* activity.

**Figure 4:**
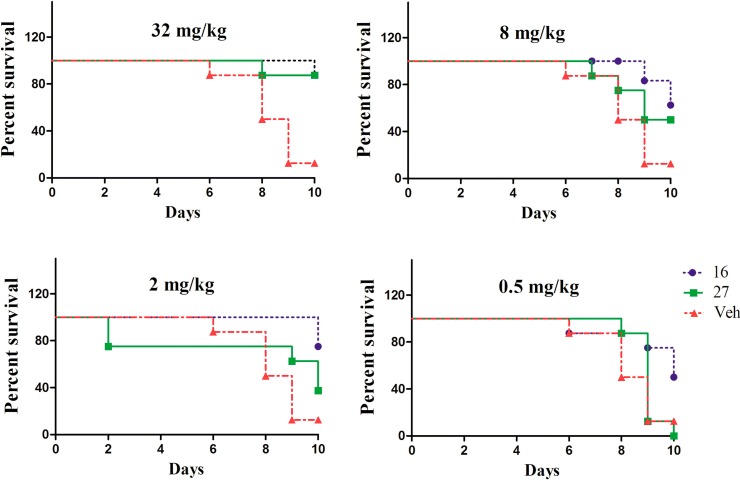
Survival rate of mice after injection of *C. albicans* 64110. FLZ stands for fluconazole and indicates a statistically significant difference compared to the control group. The four tested dosages of **31** and **42** were 32, 8, 2 and 0.5 mg/kg.

The effects of the two compounds in model mice treated with *C. albicans* 64110 (fluconazole-resistant *C. albicans* isolate) were also investigated, and the results are shown in Figure 4. Both compounds improved the survival rate of model mice, which indicated that they were effective against the fluconazole-resistant *C. albicans* isolate *in vivo*. Notably, 32 mg/kg of compound **31** kept most mice alive, displaying outstanding *in vivo* activity toward the fluconazole-resistant *C. albicans* isolate, which is promising regarding to solve clinical problems caused by fluconazole-resistant *Candida* species.

The ED_50_ of the compounds were also calculated and the results are presented in Table 3. Compound **31** was more effective toward fluconazole-resistant strain 64110 than fluconazole-susceptible isolate ATCC 90028, with ED_50_ values of 2.693 and 21.653, respectively.

**Table 3:** The ED_50_ (50% effective dose) values and 95% confidence intervals for the compounds.

Compd.	ED_50_ (mg/kg/day)	95% Confidence Interval (mg/kg/day)
**31**	21.653	2.063–227.307
	2.693	0.722–10.047
**42**	6.812	3.232–14.359
	6.944	2.698–17.871
Fluconazole	100% ^a^	

^a^ The survival rate of mice at 0.5 mg/kg dosage.

As for compound **42**, the two ED_50_ values were very similar, indicating similar activity against both strains.

## 3. Experimental Section

### 3.1. General Information

Reagents were purchased from Alfa Aesar (Shanghai, China), Acros Organics (Shanghai, China), Adamas-beta (Shanghai, China) and Shanghai Chemical Reagent Company (Shanghai, China) and were used without further purification. Analytical thin-layer chromatography (TLC) was performed using HSGF 254 plates (150–200-µm thickness, Yantai Huiyou Company, Yantai, China). Melting points were measured without correction in capillary tubes on a SGW X-4 melting point apparatus. Nuclear magnetic resonance (NMR) spectra were acquired on a Bruker AVANCE 400 NMR instrument, using TMS as an internal standard. Chemical shifts are reported in parts per million (ppm, δ) downfield from tetramethylsilane. Proton coupling patterns were described as singlet (s), doublet (d), triplet (t), quartet (q) or multiplet (m). Low- and high-resolution mass spectra (LRMS and HRMS) were acquired with electric ionization (EI) produced by a Finnigan MAT-95 spectrometer.

#### 3.1.1. General Procedure for the Synthesis of **18o**

The synthesis of **18o** was performed according to a reported method [22].

#### 3.1.2. General Procedure for the Synthesis of **18r**

Compound **18r** was synthesized according to a reported method [23].

#### 3.1.3. General Procedure for the Synthesis of Bromoacetophenone Derivatives **19**

A three-neck flask was charged with glacial acetic acid (35 mL) and the appropriate ketone (**18**, 0.02 mol). To this solution, bromine (1.1 eq) was added dropwise over 30 min at room temperature. The reaction mixture was irradiated at a power of 250 W for 5 h. The mixture was cooled, poured into 100 mL of H_2_O, and extracted three times with CH_2_Cl_2_ (60 mL). The combined organic extracts were washed with brine, dried over anhydrous sodium sulfate, filtered and evaporated under reduced pressure. The crude residue was purified by column chromatography (EtOAc/petroleum ether) to obtain the products. The data of the synthesized intermediates **19** is given in Table 4.

**Table 4:** The chemical structures, appearance, yields and ^1^H-NMR of intermediates **19**.

Compd.	Chemical Structure	Appearance Property, Yield and ^1^H-NMR
**19a**	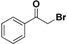	White solid; yield 72%. ^1^H-NMR (400 MHz, CDCl_3_): δ 7.99 (d, *J* = 7.7 Hz, 2H), 7.62 (t, *J* = 7.4 Hz, 1H), 7.50 (t, *J* = 7.7 Hz, 2H), 4.47 (s, 2H).
**19k**	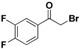	Yellow solid; yield 68.5%. ^1^H-NMR (400 MHz, CDCl_3_): δ 7.87-7.82 (m, 1H), 7.81–7.76 (m, 1H), 7.33–7.27 (m, 1H), 4.39 (s, 2H).
**19m**	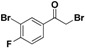	Yellow oil; yield 27%. ^1^H-NMR (400 MHz, CDCl_3_): δ 8.22 (dd, *J =* 6.5, 2.0 Hz, 1H), 7.97–7.93 (m, 1H), 7.23 (dd, *J =* 15.7, 7.5 Hz, 1H), 4.41 (s, 2H), 2.59 (s, 1H).
**19n**	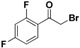	Yellow oil; yield 86%. ^1^H-NMR (500 MHz, acetone-*d*_6_): δ 8.04-7.97 (m, 1H), 7.22–7.14 (m, 2H), 4.66 (d, *J =* 2.3 Hz, 2H).
**19o**	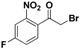	Yellow oil; yield 37.8%. ^1^H-NMR (400 MHz, CDCl_3_): δ 7.94 (dd, *J =* 8.1, 2.3 Hz, 1H), 7.60–7.49 (m, 2H), 4.30 (s, 2H).
**19q**	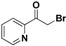	Yellow oil; yield 75%. ^1^H-NMR (400 MHz, acetone-*d*_6_): δ 8.74 (d, *J =* 4.6 Hz, 1H), 8.09–8.01 (m, 2H), 7.69 (m, 1H), 4.96 (s, 2H).
**19r**	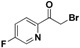	Yellow oil; yield 47.7%. ^1^H-NMR (500 MHz, CDCl_3_): δ 8.51 (d, *J =* 2.7 Hz, 1H), 8.11 (dd, *J =* 8.7, 4.7 Hz, 1H), 7.52 (m, 1H), 2.71 (s, 3H).
**19s**	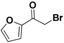	Yellow solid; yield 46%. ^1^H-NMR (400 MHz, acetone-*d*_6_): δ 7.92 (d, *J =* 0.9 Hz, 1H), 7.54–7.48 (m, 1H), 6.74 (dd, *J =* 3.6, 1.7 Hz, 1H), 4.53 (s, 2H).

#### 3.1.4. General Procedure for the Synthesis of 2-(1*H*-Imidazol-1-yl)-1-phenylethanone Derivatives **20**

Bromoacetophenone derivatives **19** (1.0 mmol) and imidazole (2.0 mmol) were dissolved in dry tetrahydrofuran (10 mL) under a nitrogen atmosphere. The reaction mixture was stirred at room temperature overnight. The solvent was removed under reduced pressure, and the residue was purified by silica gel column chromatography with dichloromethane/methanol (40:1) as the eluent to obtain the resulting intermediates. The data of the synthesized intermediates **20** is given in Table 5.

**Table 5:** The chemical structures, appearance, yields and ^1^H-NMR of intermediates **20**.

Compd.	Chemical Structure	Appearance Property, Yield and ^1^H-NMR
**20a**	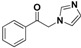	Yellow solid; yield 60%. ^1^H-NMR (400 MHz, CDCl_3_): δ 7.99 (d, *J* = 7.8 Hz, 2H), 7.67 (t, *J* = 7.4 Hz, 1H), 7.62–7.50 (m, 3H), 7.16 (s, 1H), 6.97 (s, 1H), 5.43 (s, 2H).
**20b**	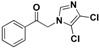	Yellow solid; yield 65%. ^1^H-NMR (400 MHz, CDCl_3_): δ 8.00 (d, *J =* 7.8 Hz, 2H), 7.70 (t, *J =* 7.4 Hz, 1H), 7.57 (t, *J =* 7.7 Hz, 2H), 7.46 (s, 1H), 5.37 (s, 2H).
**20c**	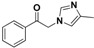	Yellow solid; yield 86%. ^1^H-NMR (400 MHz, CDCl_3_): δ 7.99 (dd, *J =* 8.4, 1.2 Hz, 2H), 7.68 (dd, *J =* 10.5, 4.4 Hz, 1H), 7.56 (d, *J =* 7.8 Hz, 2H), 7.44 (d, *J =* 0.9 Hz, 1H), 6.67 (s, 1H), 5.34 (s, 2H), 2.28 (d, *J =* 0.7 Hz, 3H).
**20d**	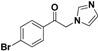	Yellow solid; yield 55%. ^1^H-NMR (400 MHz, CDCl_3_): δ 7.85 (d, *J =* 8.6 Hz, 2H), 7.69 (d, *J =* 8.6 Hz, 3H), 7.18 (s, 1H), 6.97 (s, 1H), 5.43 (s, 2H).
**20e**	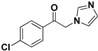	Yellow solid; yield 45.6%. ^1^H-NMR (400 MHz, CDCl_3_): δ 7.92 (d, *J* = 8.3 Hz, 2H), 7.53 (t, *J* = 8.7 Hz, 3H), 7.16 (s, 1H), 6.96 (s, 1H), 5.40 (s, 2H).
**20f**	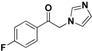	Yellow solid; yield 56%. ^1^H-NMR (400 MHz, CDCl_3_): δ 8.06-7.99 (m, 2H), 7.57 (s, 1H), 7.26–7.18 (m, 2H), 7.16 (s, 1H), 6.96 (s, 1H), 5.40 (s, 2H).
**20g**	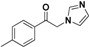	Yellow solid; yield 53.6%. ^1^H-NMR (400 MHz, CDCl_3_): δ 7.88 (d, *J* = 8.2 Hz, 2H), 7.57 (s, 1H), 7.33 (d, *J* = 8.2 Hz, 2H), 7.15 (s, 1H), 6.96 (s, 1H), 5.40 (s, 2H), 2.45 (s, 3H).
**20h**	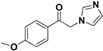	Yellow solid; yield 58.5%. ^1^H-NMR (400 MHz, CDCl_3_): δ 7.98 (d, *J =* 9.0 Hz, 2H), 7.58 (s, 1H), 7.16 (s, 1H), 7.02 (d, *J =* 9.0 Hz, 2H), 6.98 (s, 1H), 5.39 (s, 2H), 3.92 (s, 3H).
**20i**	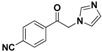	Yellow solid; yield 55.3%. ^1^H-NMR (400 MHz, CDCl_3_): δ 8.08 (d, *J =* 8.1 Hz, 2H), 7.86 (d, *J =* 8.1 Hz, 2H), 7.56 (s, 1H), 7.17 (s, 1H), 6.96 (s, 1H), 5.44 (s, 2H).
**20j**	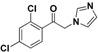	Yellow solid; yield 69.2%. ^1^H-NMR (400 MHz, CDCl_3_): δ 7.58 (d, *J =* 8.4 Hz, 1H), 7.53 (s, 1H), 7.51 (d, *J =* 1.6 Hz, 1H), 7.38 (dd, *J =* 8.4, 1.6 Hz, 1H), 7.11 (s, 1H), 6.94 (s, 1H), 5.35 (s, 2H).
**20k**	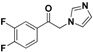	Yellow solid; yield 56%. ^1^H-NMR (400 MHz, CDCl_3_): δ 7.88-7.80 (m, 1H), 7.80–7.74 (m, 1H), 7.58 (s, 1H), 7.35 (dd, *J =* 17.0, 8.6 Hz, 1H), 7.17 (s, 1H), 6.96 (s, 1H), 5.39 (s, 2H).
**20l**	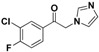	Yellow solid; yield 47.2%. ^1^H-NMR (400 MHz, CDCl_3_): δ 8.08 (dd, *J =* 6.9, 1.9 Hz, 1H), 7.95–7.87 (m, 1H), 7.76 (s, 1H), 7.32 (t, *J =* 8.5 Hz, 1H), 7.19 (s, 1H), 6.98 (s, 1H), 5.46 (s, 2H).
**20m**	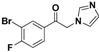	Yellow solid; yield 55.5%. ^1^H-NMR (400 MHz, CDCl_3_) δ 8.22 (dd, *J =* 6.4, 2.1 Hz, 1H), 7.96–7.92 (m, 1H), 7.56 (s, 1H), 7.28 (d, *J =* 8.1 Hz, 1H), 7.16 (s, 1H), 6.95 (s, 1H), 5.39 (s, 2H).
**20n**	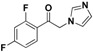	Yellow solid; yield 17.4%. ^1^H-NMR (500 MHz, acetone-*d*_6_): δ 8.10-8.06 (m, 1H), 7.56 (s, 1H), 7.33–7.20 (m, 2H), 7.10 (d, *J =* 1.0 Hz, 1H), 6.96 (s, 1H), 5.61 (d, *J =* 3.4 Hz, 2H).
**20o**	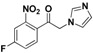	Yellow solid; yield 58.3%. ^1^H-NMR (400 MHz, CDCl_3_): δ 7.93 (dd, *J =* 8.0, 2.4 Hz, 1H), 7.57 (s, 1H), 7.64–7.59 (m, 1H), 7.44 (dd, *J =* 8.4, 5.2 Hz, 1H), 7.11 (s, 1H), 6.98 (s, 1H), 5.15 (s, 2H).
**20p**	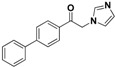	Yellow solid; yield 75%. ^1^H-NMR (400 MHz, acetone-*d*_6_): δ 8.18 (d, *J =* 8.3 Hz, 2H), 7.89 (d, *J =* 8.3 Hz, 2H), 7.78 (d, *J =* 7.7 Hz, 2H), 7.71 (s, 1H), 7.52 (d, *J =* 7.7 Hz, 2H), 7.46 (t, *J =* 7.3 Hz, 1H), 7.18 (s, 1H), 7.03 (s, 1H), 5.85 (s, 2H).
**20q**	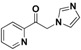	Yellow solid; yield 46.7%. ^1^H-NMR (400 MHz, CDCl_3_): δ 8.73-8.72 (m, 1H), 8.09 (d, *J =* 7.8 Hz, 1H), 7.91 (m, 1H), 7.62–7.53 (m, 2H), 7.15 (s, 1H), 6.99 (d, *J =* 1.1 Hz, 1H), 5.68 (s, 2H).
**20r**	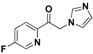	Yellow solid; yield 58.3%. ^1^H-NMR (500 MHz, CDCl_3_): δ 8.54 (d, *J =* 2.7 Hz, 1H), 8.14 (dd, *J =* 8.7, 4.6 Hz, 1H), 7.59–7.56 (m, 1H), 7.53 (s, 1H), 7.12 (s, 1H), 6.96 (s, 1H), 5.62 (s, 2H).
**20s**	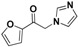	Yellow solid; yield 40%. ^1^H-NMR (400 MHz, CDCl_3_): δ 7.67 (d, *J =* 0.8 Hz, 1H), 7.55 (s, 1H), 7.29 (d, *J =* 3.6 Hz, 1H), 7.13 (s, 1H), 6.97 (s, 1H), 6.63 (dd, *J =* 3.6, 1.6 Hz, 1H), 5.25 (s, 2H).

#### 3.1.5. General Procedure for the Synthesis of **21**–**44** and **46**

To a 25-mL round-bottom flask charged with the appropriate 2-(1*H*-imidazol-1-yl)-1-phenyl-ethanone derivative **20** (1.6 mmol), (*E*)-3-(4-nitrophenyl) acrylaldehyde (1.2 eq) and toluene (4 mL) was added piperidine (55 µL) and glacial acetic acid (12 µL). The flask was evacuated, filled with nitrogen and heated to 75 °C for 4 h. The reaction mixture was cooled to room temperature and the solvent was removed. The reaction mixture was purified by silica gel column chromatography with triethylamine/petroleum ether/ethyl acetate (0.15:2:1, v/v/v) as the eluent to obtain the crude product. The crude product was washed with petroleum ether/ethyl acetate (70:1, v/v) to obtain the final product.

#### 3.1.6. Synthesis of **45**

A mixture of **31** (300 mg, 0.76 mmol) and sodium borohydride (165 mg) in absolute methanol (15 mL) was stirred at room temperature for 2 h. After removing the solvent, the residue was purified by flash column chromatography on silica gel, eluted with triethylamine/ petroleum ether/ethyl acetate (0.15:2:1, v/v/v), to afford **45** (200 mg, 66%) as yellow oil. The data of target compounds **21**–**46** are given in Table 6.

### 3.2. Biological Assays

#### 3.2.1. Antifungal Susceptibility Tests

The prepared compounds and fuconazole (Pfizer Pharmaceuticals, Shanghai, China) were dissolved in DMSO to prepare primary stocks.

The stock was then gradually diluted to prepare secondary stocks with different concentrations. Finally, the working concentrations of the derivatives were obtained by adding the appropriate amount of the secondary DMSO stocks to RPMI 1640 medium.The amount of DMSO in working solutions did not exceeded 1%. Antifungal susceptibility tests were performed according to the standard guidelines described in the Clinical and Laboratory Standards Institute document M27-A3, and the microdilution reference method was used. Next, 100 μL of RPMI 1640 medium containing the desired concentrations (128 mg/L to 0.5 mg/L) of the appropriate compound was added to each well of a 96-well plate.

**Table 6:** The chemical structures, properties, yields, ^1^H-NMR and HRMS of target compounds **21**–**46**.

Compd.	Chemical Structure	Properties	Yield, ^1^H-NMR and HRMS
**21**	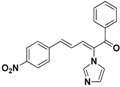	Yellow solid, mp 150–151 °C	Yield 20.8%. ^1^H-NMR (400 MHz, acetone-*d*_6_): δ 8.24 (d, *J* = 8.8 Hz, 2H), 7.89 (s, 1H), 7.81 (d, *J* = 7.6 Hz, 2H), 7.72 (d, *J* = 8.8 Hz, 2H), 7.67 (d, *J* = 7.1 Hz, 1H), 7.57 (t, *J*= 7.7 Hz, 2H), 7.48 (d, *J* = 11.1 Hz, 1H), 7.35 (s, 1H), 7.22 (s, 1H), 6.98 (dd, *J* = 15.5, 11.1 Hz, 1H). HRMS (EI) calculated for C_20_H_15_N_3_O_3_ (M^+^) 345.1106, found: 345.1113, 223.0860 (100%).
**22**	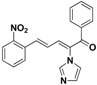	Yellow solid, mp 162–165 °C	Yield 9.7%. ^1^H-NMR (400 MHz, acetone-*d*_6_): δ 8.01 (d, *J* = 8.0 Hz, 1H), 7.84–7.73 (m, 5H), 7.68–7.61(m, 3H), 7.55 (t, *J* = 7.6 Hz, 2H), 7.49 (d, *J =* 11.1 Hz, 1H), 7.32 (s, 1H), 7.10 (s, 1H), 6.91 (dd, *J* = 15.4, 11.1 Hz, 1H). HRMS (EI) calculated for C_20_H_15_N_3_O_3_ (M^+^) 345.1113, found: 345.1110, 105.0341 (100%).
**23**	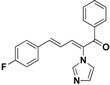	Yellow solid, mp 161–163 °C	Yield 10%. ^1^H-NMR (400 MHz, methanol-*d*_4_): δ 7.84 (s, 1H), 7.79 (d, *J =* 7.3 Hz, 2H), 7.67 (t, *J =* 7.4 Hz, 1H), 7.60–7.49 (m, 4H), 7.47 (d, *J =* 11.1 Hz, 1H), 7.31 (s, 1H), 7.26 (d, *J =* 15.5 Hz, 1H), 7.21 (s, 1H), 7.12 (t, *J =* 8.7 Hz, 2H), 6.72 (dd, *J =* 15.4, 11.1 Hz, 1H). HRMS (EI) calculated for C_20_H_15_FN_2_O (M^+^) 318.1168, found: 318.1164, 223.0870 (100%).
**24**	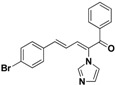	Yellow solid, mp 117–120 °C	Yield 9.5%. ^1^H-NMR (400 MHz, acetone-*d*_6_): δ 7.77 (d, *J* = 7.6 Hz, 2H), 7.72 (s, 1H), 7.66 (t, *J* = 7.4 Hz, 1H), 7.62–7.46 (m, 6H), 7.39 (d, *J* = 11.1 Hz, 1H), 7.34–7.25 (m, 2H), 7.11 (s, 1H), 6.90 (dd, *J* = 15.5, 11.1 Hz, 1H). HRMS (EI) calculated for C_20_H_15_BrN_2_O (M^+^) 378.0368, found: 378.0360, 223.0864 (100%).
**25**	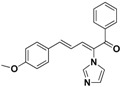	Yellow solid, mp 133–137 °C	Yield 11.4%. ^1^H-NMR (400 MHz, acetone-*d*_6_): δ 7.73 (d, *J =* 7.1 Hz, 2H), 7.67–7.59 (m, 2H), 7.53 (t, *J =* 7.5 Hz, 2H), 7.38 (dd, *J =* 15.6, 10.1 Hz, 3H), 7.19 (d, *J =* 18.7 Hz, 2H), 7.10 (s, 1H), 6.72 (d, *J =* 8.9 Hz, 2H), 6.56 (dd, *J =* 15.3, 11.3 Hz, 1H), 2.83 (d, *J =* 13.3 Hz, 3H). HRMS (EI) calculated for C_21_H_18_N_2_O_2_ (M^+^) 314.1419, found: 314.1411, 343.1678 (100%).
**26**	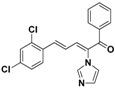	Yellow solid, mp 190–192 °C	Yield 9.8%. ^1^H-NMR (400 MHz, methanol-*d*_4_): δ 7.87 (s, 1H), 7.81 (d, *J* = 7.4 Hz, 2H), 7.68 (t, *J* = 7.5 Hz, 1H), 7.63–7.52 (m, 5H), 7.49 (d, *J* = 11.1 Hz, 1H), 7.34 (d, *J* = 13.2 Hz, 2H), 7.20 (s, 1H), 6.84 (dd, *J* = 15.5, 11.1 Hz, 1H). HRMS (EI) calculated for C_20_H_14_Cl_2_N_2_O (M^+^) 368.0483, found: 368.0474, 223.0856 (100%).
**27**	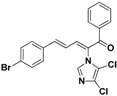	Yellow solid, mp 181–183 °C	Yield 28%. ^1^H-NMR (400 MHz, acetone-*d*_6_): δ 7.83 (d, *J =* 7.7 Hz, 3H), 7.70 (t, *J =* 7.4 Hz, 1H), 7.65 (d, *J =* 11.1 Hz, 1H), 7.63–7.55 (m, 6H), 7.42 (d, *J =* 15.5 Hz, 1H), 7.02 (dd, *J =* 15.5, 11.1 Hz, 1H). HRMS (EI) calculated for C_20_H_13_BrCl_2_N_2_O (M^+^) 445.9588, found: 445.9586, 105.0319 (100%).
**28**	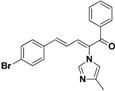	Yellow solid, mp 172–173 °C	Yield 11.3%. ^1^H-NMR (400 MHz, acetone-*d*_6_): δ 7.76 (d, *J =* 7.0 Hz, 2H), 7.65 (t, *J =* 7.4 Hz, 1H), 7.59–7.47 (m, 7H), 7.28 (dd, *J =* 15.9, 13.3 Hz, 2H), 7.01–6.92 (m, 2H), 2.20 (s, 3H). HRMS (EI) calculated for C_21_H_17_BrN_2_O (M^+^) 392.0524, found: 392.0522, 237.1022 (100%).
**29**	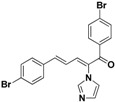	Yellow solid, mp 162–164 °C	Yield 35.4%. ^1^H-NMR (400 MHz, acetone-*d*_6_): δ 7.77–7.68 (m, 5H), 7.58 (d, *J* = 8.5 Hz, 2H), 7.49 (d, *J* = 8.5 Hz, 2H), 7.44 (d, *J* = 11.1 Hz, 1H), 7.34 (d, *J* = 15.5 Hz, 1H), 7.27 (s, 1H), 7.11 (s, 1H), 6.90 (dd, *J* = 15.5, 11.1 Hz, 1H). HRMS (EI) calculated for C_20_H_14_Br_2_N_2_O (M^+^) 455.9473, found: 455.9465, 300.9948 (100%).
**30**	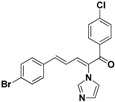	Yellow solid, mp 150–153 °C	Yield 25.8%. ^1^H-NMR (400 MHz, acetone-*d*_6_): δ 7.78 (d, *J =* 8.5 Hz, 2H), 7.71 (s, 1H), 7.58 (d, *J =* 7.1 Hz, 4H), 7.49 (d, *J =* 8.5 Hz, 2H), 7.43 (d, *J =* 11.1 Hz, 1H), 7.33 (d, *J =* 15.6 Hz, 1H), 7.27 (s, 1H), 7.11 (s, 1H), 6.90 (dd, *J =* 15.5, 11.1 Hz, 1H). HRMS (EI) calculated for C_20_H_14_BrClN_2_O (M^+^) 411.9978, found: 411.9976 257.0477 (100%).
**31**	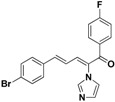	Yellow solid, mp 115–116 °C	Yield 23.4%. ^1^H-NMR (400 MHz, acetone-*d*_6_): δ 7.86 (dd, *J* = 8.7, 5.5 Hz, 2H), 7.72 (s, 1H), 7.58 (d, *J* = 8.5 Hz, 2H), 7.49 (d, *J* = 8.5 Hz, 2H), 7.41 (d, *J* = 11.1 Hz, 1H), 7.35–7.24 (m, 4H), 7.11 (s, 1H), 6.90 (dd, *J* = 15.5, 11.1 Hz, 1H). HRMS (EI) calculated for C_20_H_14_BrFN_2_O (M^+^) 396.0274, found: 396.0272, 241.0771 (100%).
**32**	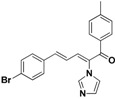	Yellow solid, mp 153–155 °C	Yield 9.5%. ^1^H-NMR (400 MHz, acetone-*d*_6_): δ 7.72–7.64 (m, 3H), 7.58 (d, *J* = 8.5 Hz, 2H), 7.48 (d, *J* = 8.5 Hz, 2H), 7.35 (dd, *J* = 9.5, 4.0 Hz, 3H), 7.32–7.23 (m, 2H), 7.10 (s, 1H), 6.90 (dd, *J* = 15.5, 11.2 Hz, 1H), 2.42 (d, *J* = 14.3 Hz, 3H). HRMS (EI) calculated for C_21_H_17_BrN_2_O (M^+^) 392.0524, found: 392.0524, 237.1025 (100%).
**33**	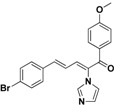	Yellow solid, mp 166–167 °C	Yield 9.5%. ^1^H-NMR (400 MHz, acetone-*d*_6_): δ 7.77 (d, *J =* 8.9 Hz, 2H), 7.71 (s, 1H), 7.58 (d, *J =* 8.5 Hz, 2H), 7.48 (d, *J =* 8.5 Hz, 2H), 7.36–7.24 (m, 3H), 7.10 (s, 1H), 7.05 (d, *J =* 8.9 Hz, 2H), 6.90 (dd, *J =* 15.5, 11.1 Hz, 1H), 3.91 (d, *J =* 7.6 Hz, 3H). HRMS (EI) calculated for C_21_H_17_BrN_2_O_2_ (M^+^) 408.0473, found: 408.0475, 253.0972 (100)%.
**34**	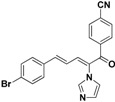	Yellow solid, mp 174–178 °C	Yield 55.3%. ^1^H-NMR (400 MHz, acetone-*d*_6_): δ 7.95 (q, *J =* 8.6 Hz, 4H), 7.72 (s, 1H), 7.58 (d, *J =* 8.5 Hz, 2H), 7.48 (dd, *J =* 9.5, 7.8 Hz, 3H), 7.34 (d, *J =* 15.5 Hz, 1H), 7.28 (s, 1H), 7.11 (s, 1H), 6.91 (dd, *J =* 15.5, 11.1 Hz, 1H). HRMS (EI) calculated for C_21_H_14_BrN_3_O (M^+^) 403.0320, found: 403.0319, 248.0824 (100%).
**35**	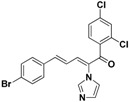	Yellow solid, mp 197–199 °C	Yield 31%. ^1^H-NMR (400 MHz, acetone-*d*_6_): δ 7.66 (d, *J* = 12.3 Hz, 3H), 7.57 (d, *J* = 8.2 Hz, 3H), 7.47 (d, *J* = 8.1 Hz, 2H), 7.35 (dd, *J* = 21.5, 13.3 Hz, 2H), 7.25 (s, 1H), 7.13 (s, 1H), 6.87 (dd, *J* = 15.1, 11.3 Hz, 1H). HRMS (EI) calculated for C_20_H_13_BrCl_2_N_2_O (M^+^) 445.9588, found: 445.9588, 291.0083 (100%).
**36**	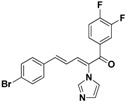	Yellow solid, mp 132–133 °C	Yield 74.9%. ^1^H-NMR (400 MHz, acetone-*d*_6_): δ 7.78–7.70 (m, 2H), 7.65 (s, 1H), 7.58 (d, *J* = 8.4 Hz, 2H), 7.50 (t, *J* = 8.9 Hz, 4H), 7.34 (d, *J* = 15.5 Hz, 1H), 7.28 (s, 1H), 7.11 (s, 1H), 6.90 (dd, *J* = 15.5, 11.1 Hz, 1H). HRMS (EI) calculated for C_20_H_13_BrF_2_N_2_O (M^+^) 414.0179, found: 414.0177, 259.0675. (100%).
**37**	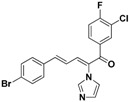	Yellow solid, mp 111–112 °C	Yield 52.1%. ^1^H-NMR (400 MHz, acetone-*d*_6_): δ 7.91 (dd, *J =* 7.2, 2.1 Hz, 1H), 7.80–7.77 (m, 1H), 7.73 (s, 1H), 7.58 (d, *J =* 8.5 Hz, 2H), 7.49 (dd, *J =* 13.0, 4.7 Hz, 4H), 7.34 (d, *J =* 15.6 Hz, 1H), 7.28 (s, 1H), 7.11 (s, 1H), 6.90 (dd, *J =* 15.5, 11.1 Hz, 1H). HRMS (EI) calculated for C_20_H_13_BrClFN_2_O (M^+^) 429.9884, found: 429.9883, 275.0367 (100%).
**38**	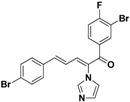	Yellow solid, mp 133–134 °C	Yield 89%. ^1^H-NMR (400 MHz, acetone-*d*_6_): δ 8.04 (dd, *J =* 6.7, 2.1 Hz, 1H), 7.84–7.80 (m, 1H), 7.73 (s, 1H), 7.58 (d, *J =* 8.5 Hz, 2H), 7.49 (d, *J =* 8.8 Hz, 3H), 7.44 (d, *J =* 8.6 Hz, 1H), 7.34 (d, *J =* 15.6 Hz, 1H), 7.29 (s, 1H), 7.11 (s, 1H), 6.90 (dd, *J =* 15.5, 11.1 Hz, 1H). HRMS (EI) calculated for C_20_H_13_Br_2_FN_2_O (M^+^) 473.9379, found: 473.9381, 318.9905 (100%).
**39**	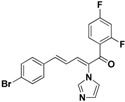	Yellow solid, mp 148–150 °C	Yield 55.1%. ^1^H-NMR (500 MHz, acetone-*d*_6_): δ 7.79–7.72 (m, 1H), 7.69 (s, 1H), 7.59 (d, *J* = 8.5 Hz, 2H), 7.48 (dd, *J* = 13.8, 9.9 Hz, 3H), 7.34 (d, *J* = 15.5 Hz, 1H), 7.25–7.17 (m, 3H), 7.11 (s, 1H), 6.90 (dd, *J* = 15.5, 11.1 Hz, 1H). HRMS (EI) calculated for C_20_H_13_BrF_2_N_2_O (M^+^) 414.0179, found: 414.0166, 259.0647 (100%).
**40**	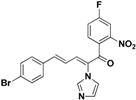	Yellow solid, mp 183–184 °C	Yield 52.3%. ^1^H-NMR (500 MHz, acetone-*d*_6_): δ 8.12 (dd, *J* = 8.6, 2.5 Hz, 1H), 7.88 (dd, *J* = 8.5, 5.4 Hz, 1H), 7.80–7.83 (m, 1H), 7.65 (s, 1H), 7.56 (d, *J* = 8.5 Hz, 2H), 7.45 (d, *J* = 8.5 Hz, 2H), 7.39 (d, *J* = 11.0 Hz, 1H), 7.26–7.21 (m, 2H), 7.12 (s, 1H), 6.83 (dd, *J* = 15.5, 11.1 Hz, 1H). HRMS (EI) calculated for C_20_H_13_BrFN_3_O_3_ (M^+^) 441.0124, found: 441.0130, 288.9987 (100%).
**41**	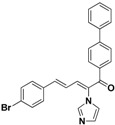	Yellow solid, mp 167–168 °C	Yield 52.3%. ^1^H-NMR (400 MHz, acetone-*d*_6_): δ 7.90 (d, *J =* 8.3 Hz, 2H), 7.85 (d, *J =* 8.3 Hz, 2H), 7.78 (d, *J =* 6.7 Hz, 4H), 7.66–7.50 (m, 5H), 7.46 (d, *J =* 7.3 Hz, 1H), 7.42 (d, *J =* 8.6 Hz, 1H), 7.32 (s, 1H), 7.12 (s, 1H), 6.98 (dd, *J =* 15.5, 11.1 Hz, 1H). HRMS (EI) calculated for C_26_H_19_BrN_2_O (M^+^) 454.0681, found: 454.0683, 299.1186 (100%).
**42**	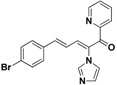	Yellow solid, mp 114–116 °C	Yield 6.5%. ^1^H-NMR (400 MHz, acetone-*d*_6_): δ 8.72 (d, *J* = 4.4 Hz, 1H), 8.13 (d, *J* = 11.2 Hz, 1H), 8.05 (t, *J* = 7.1 Hz, 1H), 7.94 (d, *J* = 7.8 Hz, 1H), 7.69–7.62 (m, 2H), 7.58 (d, *J* = 8.4 Hz, 2H), 7.49 (d, *J* = 8.4 Hz, 2H), 7.33 (d, *J* = 15.6 Hz, 1H), 7.21 (s, 1H), 7.10 (s, 1H), 6.84 (dd, *J* = 15.6, 11.2 Hz, 1H). HRMS (EI) calculated for C_19_H_14_BrN_3_O (M^+^) 379.0320, found: 379.0318, 224.0823 (100%).
**43**	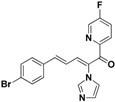	Yellow solid, mp 156–157 °C	Yield 4.3%. ^1^H-NMR (500 MHz, acetone-*d*_6_): δ 8.66 (d, *J =* 2.8 Hz, 1H), 8.16–8.08 (m, 2H), 7.93–7.89 (m, 1H), 7.69 (s, 1H), 7.62 (d, *J =* 8.5 Hz, 2H), 7.52 (d, *J =* 8.5 Hz, 2H), 7.36 (d, *J =* 15.6 Hz, 1H), 7.24 (s, 1H), 7.13 (s, 1H), 6.88 (dd, *J =* 15.6, 11.2 Hz, 1H). HRMS (EI) calculated for C_19_H_13_BrFN_3_O (M^+^) 397.0226, found: 397.0227, 242.0732 (100%).
**44**	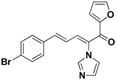	Yellow solid, mp 180–182 °C	Yield 21.1%. ^1^H-NMR (400 MHz, acetone-*d*_6_): δ 7.90 (d, *J* = 0.9 Hz, 1H), 7.78 (d, *J* = 11.2 Hz, 1H), 7.74 (s, 1H), 7.59 (d, *J* = 8.5 Hz, 2H), 7.49 (d, *J* = 8.5 Hz, 2H), 7.40 (d, *J* = 15.6 Hz, 1H), 7.29 (s, 1H), 7.17 (s, 1H), 6.78 (dd, *J* = 15.6, 11.2 Hz, 1H), 6.65 (dd, *J* = 3.6, 1.6 Hz, 1H), 6.57 (d, *J* = 3.5 Hz, 1H). HRMS (EI) calculated for C_18_H_13_BrN_2_O_2_ (M^+^) 368.0160, found: 368.0157, 213.0664 (100%).
**45**	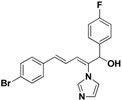	Yellow oil	Yield 66%. ^1^H-NMR (400 MHz, acetone-*d*_6_): δ 7.50 (d, *J* = 8.5 Hz, 2H), 7.40 (s, 1H), 7.36–7.32 (m, 4H), 7.04 (t, *J* = 8.8 Hz, 2H), 6.99 (s, 1H), 6.94 (s, 1H), 6.89 (d, *J* = 15.7 Hz, 1H), 6.81 (d, *J* = 11.0 Hz, 1H), 6.57 (dd, *J* = 15.7, 10.9 Hz, 1H), 5.64 (d, *J* = 8.2 Hz, 1H).HRMS (EI) calculated for C_20_H_16_BrFN_2_O (M^+^) 398.0430, found: 398.0424, 243.0927 (100%).
**46**	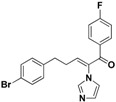	Yellow oil	Yield 20.1%. ^1^H-NMR (500 MHz, acetone-*d*_6_): δ 7.76 (dd, *J* = 8.5, 5.6 Hz, 2H), 7.48 (d, *J* = 8.2 Hz, 3H), 7.26 (t, *J* = 8.7 Hz, 2H), 7.17 (d, *J* = 8.1 Hz, 2H), 7.03 (d, *J* = 10.1 Hz, 2H), 6.74 (t, *J* = 7.4 Hz, 1H), 2.86 (t, *J* = 7.3 Hz, 2H), 2.58 (q, *J* = 7.4 Hz, 2H). HRMS (EI) calculated for C_20_H_16_BrFN_2_O (M^+^) 398.0430, found: 398.0433, 123.0243 (100%).

Eight strains of *Candida* spp. were used in the assay, including the quality control *Candida albicans* ATCC 90028 and *Candida krusei* ATCC 6528 isolates. They were cultured in solid Yeast Extract Peptone Dextrose (YPD) medium at 37 °C in a humidified atmosphere of 5% CO_2_ in air. The cells were dissolved in normal saline at a density of 5 × 10^6^ CFU/mL. Then, the solution was diluted 1000 times with RPMI 1640 medium, and 100 μL of diluted solution was added to the 96-well plate containing the compounds. After incubation for 48 h at 37 °C, the MIC was read as the lowest concentration that produced a prominent decrease in growth (inhibition ≥ 80%) compared to the control cells (without compound).

#### 3.2.2. Antibacterial Susceptibility Tests

The microdilution reference method was performed according to the standard guidelines described in the Clinical and Laboratory Standards Institute document M07-A9 for the antibacterial susceptibility assays. The desired working concentrations of the derivatives **31**, **42**, and four positive drugs (amikacin, cefoperazone, vancomycin, and erythromycin) were obtained by adding the secondary DMSO stocks to broth culture, as described for the antifungal susceptibility assays. Then, 100 μL of broth culture containing the appropriate concentrations (128 mg/L to 0.5 mg/L) of each compound was added to each well of a 96-well plate.

Eight strains of bacteria were used: Gram-positive bacterial isolates *Staphylococcus aureus* UA1758, *Staphylococcus epidermidis* UF843, and *Enterococcus faecalis* UA257, and Gram-negative bacterial isolates *Klebsiella pneumonia* UF222, *Escherichia coli* UA45, ESBL-producing *Escherichia coli*, *Acinetobacter baumannii* UA1037, and *Pseudomonas aeruginosa* UA1024. After culture in a blood plate at 37 °C in a humidified atmosphere of 5% CO_2_ in air, cells were suspended in normal saline at a density of 2 × 10^8^ CFU/mL. Then, the solution was diluted 1000 times with culture broth, and 100 μL of diluted solution was added to the 96-well plate containing compounds. After incubation for 24 h at 37 °C, the MIC was read as the lowest concentration that produced a prominent decrease in growth (100% inhibition) compared with the control cells (without compound).

#### 3.2.3. Mice Toxicity Assays

Sixteen mice (equal numbers of males and females) were selected to test the toxicity of compound **31** in mice. They were randomly grouped into four groups (four mice in each group): 32 mg/kg group, 16 mg/kg group, 8 mg/kg group and control group. Each group of mice was given compound **31** by intragastric administration consecutively for 5 days with dosages of 32, 16 and 8 mg/kg once a day. The mice in the control group were given 0.5% sodium carboxymethyl cellulose solution at 20 mL/kg. The survival of the mice was recorded

#### 3.2.4. *In Vivo* Antifungal Activity

To establish a systemic fungal infection model, 160 Kunming mice, half males and half females, were selected, with weights ranging from 18 to 22 g and that had passed the quarantine inspection. They were divided into four groups according to their weight: compound **31** strain No. 1 group (I), compound **31** strain No. 2 group (II), compound **42** strain No. 1 group (III) and compound **42** strain No. 2 group (IV). Each group contained equal numbers of males and females. The 40 mice in group I containing half males and half females were divided into four dose groups and a control group, with 8 mice in each group, containing four of each sex. The mice in each dose group were given a different dosage (0.5, 2, 8 or 32 mg/kg) of compound **31** by intragastric administration, and the mice in the control group were given 0.5% sodium carboxymethyl cellulose solution at 20 mL/kg. A concentration of 2 × 10^6^ CFU/mL of strain No. 1 was given by tail vein injection approximately 0.5 h after intragastric administration of the compound. Drugs were administered to mice once a day for 10 consecutive days. The survival rate and ED_50_ were calculated for each group. The remaining groups (II, III and IV) were given the relevant drugs and strains according to the group designations presented above. The test method was similar to what is described above. Testing of fluconazole (positive control) was performed according to the method similar to that described above.

Result processing: the ED_50_ and 95% confidence limit were calculated for each group using the regular Bliss method, and a graph of the survival curve was plotted based on the relationship between the survival rates of the mice over time.

## 4. Conclusions

In conclusion, we observed inhibitory activity of compound **24** containing an imidazole moiety and a 2,4-dienone motif oward several fungi. Based on this, a total of 26 derivatives were designed and synthesized in three steps. The prepared compounds were tested using *in vitro* antifungal activity assays, and several compounds exhibited improved antifungal activities compared to the lead compound. Among these compounds, compounds **31** and **42** exhibited strong inhibitory effects toward *Candida* species. In particular, the two derivatives exhibited potent antifungal activities toward the fluconazole-resistant isolate *C. albicans* 64110, with both having MIC values of 8 µg/mL. In addition, they displayed obvious effects against two Gram-positive bacteria, *Staphylococcus epidermidis* UF843 and *Staphylococcus aureus* UA1758. The results of animal experiments indicated that both compounds could improve the survival rate of model mice treated with ATCC 90028 (fluconazole-susceptible isolate). More importantly, the two compounds exhibited outstanding effects toward a fluconazole-resistant *C. albicans* isolate *in vivo*, which is promising with regard to the clinical problem posed by fluconazole-resistant *Candida* species.
